# Scabies outbreak investigation and risk factors in Kechabira district, Southern Ethiopia: unmatched case control study

**DOI:** 10.1186/s13104-019-4317-x

**Published:** 2019-05-29

**Authors:** Wondimu Wochebo, Yusuf Haji, Solomon Asnake

**Affiliations:** 1 Ethiopia Field Epidemiology Training Program, Addis Ababa, Ethiopia; 2 School of Public Health, Hawassa University, Hawassa, Ethiopia; 3 School of Medical laboratory Sciences, Hawassa University, Hawassa, Ethiopia

**Keywords:** Scabies, Outbreak investigation, Hobichaka, Kechabira district

## Abstract

**Objective:**

Scabies is an infection of the skin, which caused by human itch mite *Sarcoptes scabiei*. It is a common health problem in Ethiopia, especially during disasters, poor sanitation and overcrowded living condition. However, investigation on scabies outbreak and associated factors was absent or scarce in the country in general and in the study area in particular. Hence, this study was intended to investigate scabies outbreak, identify risk factors, and recommend preventive measures in Kechabira district, Kembata Tembaro zone, Southern Ethiopia.

**Result:**

We identified a total of 243 scabies cases line listed with overall prevalence of 2.5% and attack rate of (AR) 20.5 per 1000 populations and no death was reported. Of the suspected cases 126 (51.9%) were males and 117 (48.1%) were females. The median age was 24 years with inter-quartile range (IQR) of 22 years. The highest cases were seen in children aged 5–14 (50.6%) years. The cases were seen in three villages and the highest incidence was in Burchana, 23.9 per 1000 population. Identified determinant factors for scabies outbreak were sharing clothes with scabies patients (AOR = 6.08, 95% CI [1.54–23.92], and households having greater than six family members AOR = 38.755, 95% CI [8.084–185.787].

**Electronic supplementary material:**

The online version of this article (10.1186/s13104-019-4317-x) contains supplementary material, which is available to authorized users.

## Introduction

Scabies is a neglected tropical parasitic disease that is a major public health problem worldwide, and particularly in resource-poor regions. As WHO report, 2018 indicated scabies is a common public health problem that affects about 200 million people globally, with an estimated prevalence that range from 0.2 to 71% [1]. It is a contagious skin infestation caused by infection with the female mite *Sarcoptes scabiei* *var.* *hominis* [2]. Overcrowding, poor hygiene, poor nutritional status, immigration, homelessness and sexual contact are the common predisposing factors for the infestation [3]. Studies in Sub-Saharan African countries indicated that scabies is highly contagious skin infection [4] and spreads by direct, prolonged, skin-to-skin contact with an infested individual and cause debilitating itching, leading to scratching and expose to secondary bacterial infection [5]. Most affected are children at the age of school [6] particularly children in institutional environments and closed communities experience high endemic rates and epidemic outbreaks in tropical and developing countries [7, 8]. Scabies outbreak occurs in many parts of Ethiopia as public health problem being beyond sporadic and affecting wider geographic areas and population [9]. Study conducted in Ethiopia, Amhara region indicated that the scabies prevalence in the 68 districts ranged from 2 to 67% with a median prevalence of 33.5 [3]. Study conducted in a district South region also indicated a prevalence of 11% [9]. Kechabirra is one of the scabies outbreak affected district. Therefore, this study was designed to investigate scabies outbreak, confirm the occurrence of scabies, identify the risk factors and suggest practical prevention and control measures to alleviate the disease burden of the community.

## Main text

### Methods

#### Study area and design

Unmatched community based case- control and descriptive studies were conducted from July 8 to 24, 2017 at Kechabirra district. Kechabirra district was found in Kembata Tembaro zone, SNNPR, 358 km from Addis Abeba, and 139 km from Hawassa. The total population of the district was 124,058, male (60,788) and female (63,270) with 25,318 households and the average in habitants was estimated to be 4.9/households and under five ages children were 19,365 for 2016/17 [10] (Additional file 1).

#### Data collection methods and analysis

Total sample size was estimated using Epi Info Stat Calc version 7 at confidence level of 95%, margin of error of 5% and power of 80% and 10% non response rate. The collected data include sociodemographic characteristics, clinical features and management of the cases and the possible risk factors. Data was collected using face-to-face interview, line lists data (the list of scabies suspected cases obtained from house to house survey for search of scabies suspected cases during the outbreak investigation period) and key informant interview techniques and analyzed using SPSS version 20 software. P value < 0.05 was considered as statistically significant.

### Results

#### Descriptive epidemiology

From July 8–24, 2017 we identified a total of 243 suspected scabies cases that line listed from three villages of the district. The overall prevalence of scabies was 2.5 and the attack rate (AR) in the three villages was 20.5 per 1000 populations with no scabies related death (CFR = 0). Of the total suspected scabies cases, 126 (51.9%) were males and 117 (48.1%) were females and sex specific attack rate (SSAR) for females was 117 (18.5/1000) and male 126 (20.7/1000). Age-specific attack rate (ASAR) was highest among the age group of Children of 5–14 years of age with an attack rate of 11/1000 population (Table 1).

**Table 1:** Sociodemographic Status of suspected scabies cases in Kechabira district, Southern Ethiopia

Variables	Category	Number	Percent (%)
Sex	Male	126	51.9
	Female	117	48.1
Age (years)	0–4	21	8.6
	5–14	123	50.6
	15–45	85	35
	> 45	14	5.8
Address (village)	Hobichaka	86	35.4
	Doreba	79	32.5
	Burichana	78	32.1
Religion	Protestant	189	77.7
	Orthodox	40	16.5
	Catholic	14	5.8
Occupation	Student	141	58.2
	Farmer	75	30.8
	Others	27	11
Marital status	Married	26	10.7
	Not married	217	89.3
Educational status	Formal	183	75.3
	Non-formal (not attend school)	60	24.7
Family member	< 6	206	84.8
	≥ 6	37	15.2

**Fig. 1:**
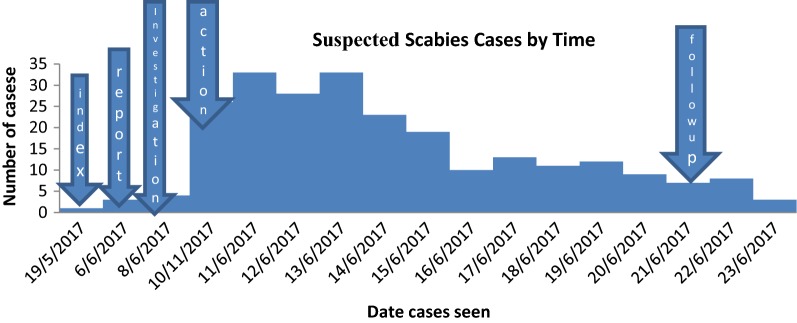
Epidemic curve of scabies outbreak in Hobichaka cluster, Kechabira district, Southern Ethiopia

The index case of the outbreak was observed in Doreba village on 12 year old child on May 19, 2017. Kechabira district reported the suspected scabies to zonal health department on July 6/2017. The zonal public health emergency team sent the investigation team to district and the team confirmed the existence of scabies cases from July 8–9, 2017 (Fig. 1).

#### Case control analysis

A total of 123 study participants (41 cases 82 controls with case to control ratio of 1:2) were purposively selected to identify risk factors for scabies outbreak from affected three villages. The age of study participants range from 5 to 65 years, the mean age was 24.8 with (Std. 13.274). The proportion of male study subjects were 54 (43.9%) while females were 69 (56.1%). All the cases had skin rashes 41 (100%), (90%) had red bumps and blister; 92% presented with itching sensation at night and none of the cases have sign of secondary infection (Additional file 2). Regarding site of rash almost all (92.7%) had rash on their finger and least (40%) on the anterior axillary and peds (Additional file 3).

Concerning risk factors, after adjusting for possible confounding factors, showering less than two times per week [AOR = 11.62, 95% CI (2.972–45.418)], sharing clothes with scabies patients [AOR = 6.083, 95% CI (1.546–23.927)] poor hand washing, [AOR = 5.155, 95% CI (1.286–20.666)], family members greater than six [AOR = 38.755, 95% CI (8.084–185.785)] and infrequent use of soap AOR = [4.777, 95% CI 1.440, 15.841)] were independent risk factors associated with scabies outbreak (Table 2).

**Table 2:** Bivariate and multivariate logistic regression analysis of scabies outbreak and its determinant factors in Kechabira district, Southern Ethiopia

Variables	Categories		COR (95% CI)	AOR (95% CI)	P-value
	Case	Control			
Being low educated					
Yes	18	20	2.42 [1.09–5.38]	1.44 [0.41–5.06]	0.57
No	23	62	1	1	
Skin contact with scabies cases within 2 months					
Yes	25	29	2.85 [1.32–6.19]	0.79 [0.22–2.75]	0.71
No	16	53	1	1	
Bathing < 2×/week					
Yes	24	18	5.02 [2.23–11.30]	9.77 [2.44–39.09]	0.001
No	17	64	1	1	
Poor hand washing practice					
Yes	18	18	2.78 [1.24–6.24]	5.76 [0.312–25.30]	0.02
No	23	64	1	1	
Changing clothes					
Yes	20	23	2.44 [1.12–5.32]	2.07 [0.66–6.78]	0.22
No	21	59	1	1	
Family member > six living together					
Yes	25	12	9.11 [3.79–21.90]	38.21 [7.72–189.08]	0.000
No	16	70	1	1	
Sharing cloth with scabies patient					
Yes	15	13	3.06 [1.28–7.30]	5.73 [1.26–26.06]	0.02
No	26	69	1	1	
Infrequent use of soap					
Yes	22	22	3.15 [1.44–6.92]	4.69 [1.34–16.36]	0.01
No	19	60	1	1	

### Discussion

The overall prevalence rate of scabies was 2.5%, the prevalence of this investigation was comparable with findings of investigations conducted in Kuwait [11], Egypt (4.4) [12], and Nigeria (4.8) [13], but higher than what has been reported from Iran in 2008 (1.7%) [14]. Our result is lower than what was reported from North western Ethiopia Amhara region where the median prevalence was 33.5% [3], Gonder 22.5% [15], Southern Ethiopia 11% [9], Cameroon 18.5% [16], Nigeria 10.5% [17], Sierra Leone (67%) [8], Solomon Island (19.5) [18], Malaysia (31%) [5], Bangladesh (62%) [19]. As compared to the above findings the prevalence in our case was lower, which might be associated with the study condition, which was held at community level, while the above investigations were conducted in institutions. As well it was known although scabies can affect individuals at any socioeconomic level; individuals who live in overcrowded conditions are at much higher risk for scabies [20].

The attack rate of scabies among both sexes in our case was nearly the same, our finding was in accordance with that of [3, 5]. However, study conducted in Southern Ethiopia [9], in Palestine [21], in Fuji [22] and Iran [14] indicated that males are more infested than females. While, study conducted in Nigeria [13] and Cameroon [16] indicated more than half of the scabies cases were females than males. This scabies infestation occurs in any age subgroups, but commonly seen in younger ages. Particularly the most affected age group were those in the age group of 5–14 with an attack rate of 131/1000 population. Analogous findings were recorded by investigations conducted in Ethiopia [9], Fuji [22], Nigeria [13] and Cameroon [16] where the school-aged children commonly affected. The possible reasons for wide spread of scabies among young children could be the close contact among peers, overcrowding in schools and sharing of contaminated private materials particularly closes.

With reference to the sites of lesions, inter-digital spaces, flexor surfaces of the wrists, buttocks, elbows and genitalia were the scabies infested sites. Most of the cases have skin rashes in the beginning of symptoms and had red bumps and blister on their finger webs presented with itching sensation at night. The findings are in line with study conducted in Southern Ethiopia [9], Iran [14], and Cameroon [16]. This might be due to these sites are hide and delicate and preferable for survival and perpetuation and infestation of the ecto-parasite. Hence, the infestation persist long actually not reach to life-threatening condition but can be severe and leading to debilitation, discomfort, depression, and secondary skin infections [21].

Regarding the risk factors associated with scabies, there is a statistically significant association between level of education and scabies infestation, and hence the risk of developing scabies is two times more in low educated individuals compared to their counterparts. The present work was in accordance with findings of outbreak investigation of scabies conducted in Northern Ethiopia Tigray [23]. Moreover, Feldmeier and Heukelbach [24] and Ursani and Baloch [25] declared that illiteracy and low standard of education are the factor responsible for the distribution of scabies. This might be due to the fact that people who are of lower educational level are less aware of personal hygiene rules to adopt especially when living with others, therefore, they might be more prone to be infected.

Skin contact also has significant association with scabies infestation; hence the odds of developing scabies was three times more in those individuals who reported that they have contact within the last 2 months with scabies patient as compared to those who did not have such contact. Our finding was in line with study conducted in Tigray, Northern Ethiopia, indicated that getting scabies was five times more in clients having physical contact with scabies patient [23]. Similar findings were also obtained in systemic review conducted in developing countries, which indicated having skin contact with a person infested with scabies in the past 2 months was risk factors for scabies [5].

The risk of getting scabies is also strongly associated with personal hygiene, thus those who mentioned that they took bathing less than two times per week were five times more likely develop scabies compared to their counterparts. Our study was in accordance with study conducted in North western Ethiopia, Gonder [15], where individuals who wash their body in more than a week interval were 3.22 times more likely develop scabies. Similarly, in Northern Ethiopia (Tigray) [23], the risk of getting scabies was five times more in individuals having poor personal hygiene particularly poor hand washing practices. In contrast, different studies indicated that the prevalence of scabies was not influenced by personal hygiene [26, 27]. Experimental findings also showed that viability and number of *Sarcoptes scabiei* was not reduced by hand washing or rubbing hands with alcohol [28]. Scholars sagest such over guessing view might occur due to false impression that substance that are bactericidal or virucidal will be effective against mites [29].

In this study family size has statistically significant association with scabies infestation, in those participants having family size more than five, the odds of acquiring scabies was higher as compared to those reporting less than five persons per households. Our finding was in line with investigation conducted in Northern Ethiopia Tigray region [23] where households having more than six individuals were nine times more likely to acquire scabies as compared to those having less than 5. Similar findings was recorded in Southern Ethiopia [9] where households with family size more than five are 2.6 times more at risk of harboring scabies. Result from Solomon Islands as well indicated family size having six to ten members was 1.4 times more likely to acquire scabies as compared to those households having less than 5 family members [23]. Study performed in Iran as well designated that family size was directly associated with scabies infestation [14]. The positive association of larger family size with scabies infestation might be due to overcrowding among larger family members compared to the smaller family size, which augments access of sharing of bed, cloths, and other materials that might transmit the infection, since scabies can spread easily under crowded conditions where close body and skin contact is common [30].

### Conclusions

There is a scabies outbreak occurred in Hobichaka cluster, Kechabira district, KT zone SNNPR. The overall attack rate of the outbreak is high. Kechabira district is too late to identify public health problem of scabies. Both sharing of clothes with scabies patients, Contact with scabies patient and infrequent changing of clothes were major risk factors for the occurrence outbreak in the area. There is limited access and shortage of water in the area which contribute positively in personal hygiene and washing. Hence, delaying in management of suspected cases should strengthen and active surveillance should be started at all clusters. Health education should be given to improve the awareness of community in prevention and control of scabies and access to safe water should be improved.

## Limitation of study

Since our study was carried out based on clinical signs and symptoms without laboratory confirmatory test, there was a problem of determination of cases. In addition in view of the fact that we didn’t employ microscopic test we face a problem in determination of crusts, presence of burrows and secondary bacterial infections. Moreover given that our study design was a case control type the role of recall bias could not be ruled out.

## Additional files

**Additional file 1.** Map of Hobichaka cluster, Kechabira district, Kembata Tembaro zone, Southern Ethiopia.

**Additional file 2.** Cases with clinical features of scabies, Hobichaka cluster, Kechabira district, Southern Ethiopia.

**Additional file 3.** Site of the rash on the body of cases, Hobichaka cluster, Kechabira district, Southern Ethiopia.

## Data Availability

The datasets supporting the conclusions of this article are included within the article.

## Abbreviations

SNNPR: South Nation Nationalities People Region
FMoH: Federal Minster of Health
WHO: World Health Organization
CFR: scabies related death
SSAR: sex specific attack rate
ASAR: age-specific attack rate
