# Supplementary material for: Scabies outbreak investigation and risk factors in Kechabira district, Southern Ethiopia: unmatched case control study
Source: BMC Res Notes. 2019 May 29;12:305. doi: 10.1186/s13104-019-4317-x (PMC6542071; doi:10.1186/s13104-019-4317-x)
Supplement: Supplementary file 1 — Additional file 1. Map of Hobichaka cluster, Kechabira district, Kembata Tembaro zone, Southern Ethiopia. [file 13104_2019_4317_MOESM1_ESM.docx]

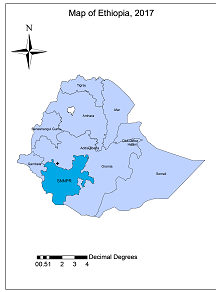

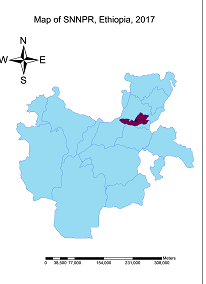


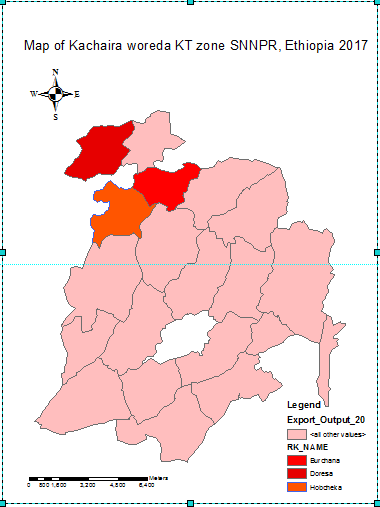

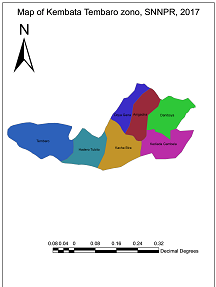


Additional file 1. Map of Hobichaka cluster, in Kechabira district, Kembata Tembaro zone, Southern Ethiopia. (ArcGIS software, version 10, www.esri.com)
