# Supplementary material for: Scabies outbreak investigation and risk factors in Kechabira district, Southern Ethiopia: unmatched case control study
Source: BMC Res Notes. 2019 May 29;12:305. doi: 10.1186/s13104-019-4317-x (PMC6542071; doi:10.1186/s13104-019-4317-x)
Supplement: Supplementary file 2 — Additional file 2. Cases with clinical features of scabies, Hobichaka cluster, Kechabira district, Southern Ethiopia. [file 13104_2019_4317_MOESM2_ESM.docx]

Additional file 2. Cases with clinical features of scabies, Hobichaka cluster, Kechabira Distict, Southern Ethiopia
